# Biallelic *SORD* pathogenic variants cause Chinese patients with distal hereditary motor neuropathy

**DOI:** 10.1038/s41525-020-00165-6

**Published:** 2021-01-04

**Authors:** Hai-Lin Dong, Jia-Qi Li, Gong-Lu Liu, Hao Yu, Zhi-Ying Wu

**Affiliations:** 1 Department of Neurology and Research Center of Neurology in Second Affiliated Hospital, and Key Laboratory of Medical Neurobiology of Zhejiang Province, Zhejiang University School of Medicine, Hangzhou, China; 2 Department of Neurology and Institute of Neurology, Huashan Hospital, Shanghai Medical College, Fudan University, Shanghai, China

**Keywords:** Molecular medicine, Peripheral neuropathies

## Abstract

Sorbitol dehydrogenase gene (*SORD*) has been identified as a novel causative gene of recessive forms of hereditary neuropathy, including Charcot–Marie–Tooth disease type 2 and distal hereditary motor neuropathy (dHMN). Our findings reveal two novel variants (c.404 A > G and c.908 + 1 G > C) and one known variant (c.757delG) within *SORD* in four Chinese dHMN families. Ex vivo cDNA polymerase chain reaction confirmed that c.908 + 1 G > C variant was associated with impaired splicing of the SORD transcript. In vitro cell functional studies showed that c.404 A > G variant resulted in aggregate formation of SORD and low protein solubility, confirming the pathogenicity of *SORD* variants. We have provided more evidence to establish *SORD* as a causative gene for dHMN.

The distal hereditary motor neuropathy (dHMN) is a group of clinically and genetically heterogeneous inherited peripheral neuropathy, characterized by slowly progressive distal limb muscle weakness and atrophy, tendon areflexia, and foot deformities^1^. The dHMN often shares a phenotypic overlap with the axonal form of Charcot-Marie-Tooth disease type 2 (CMT2) with no or minor sensory neuropathy. In addition, it has an overlap with CMT2 in causative genes, such as *GARS*, *IGHMBP2*, *AARS*, and *HARS* genes^2,3^. Although at least 30 disease-causing genes for dHMN have been identified (http://neuromuscular.wustl.edu), the genetic diagnosis remains elusive for 70–80% of affected individuals^4,5^.

Sorbitol dehydrogenase gene (*SORD*) encodes a 357 amino acid protein and the protein acts as a key enzyme in the polyol pathway. Very recently, variants in *SORD* have been identified as a novel cause, and also the most common form in individuals affected by CMT2 or dHMN^6^. Totally, 45 affected individuals from 38 families, including 23 CMT2 (3 from China), 18 dHMN, and 4 intermediate CMT patients were identified to carry homozygous or heterozygous c.757delG (p.Ala253GlnfsTer27) variant in *SORD*.

Here, we report the identification of two novel variants (c.404 A > G and c.908 + 1 G > C) and one known variant (c.757delG) in *SORD* in four Chinese dHMN families. This report further establishes *SORD* as a disease-causing gene for dHMN, and expands both the phenotypic and mutational spectra of the SORD-associated hereditary neuropathy.

To investigate the underlying causative gene in Chinese CMT2 and dHMN, we have collected a cohort of 20 CMT2 and 9 dHMN patients without known genetic cause. Whole-exome sequencing (WES) was conducted on all the patients. After analyzing and filtering, we found three dHMN patients from family 1, 2, and 3 carrying the known homozygous c.757delG (p.Ala253GlnfsTer27) variant in *SORD*. Furthermore, two novel variants were detected in the proband (dHMN) of family 4, including a missense variant c.404 A > G (p.His135Arg) and a splicing variant c.908 + 1 G > C. The results of Sanger sequencing confirmed the presence of the identified variants in corresponding individuals and showed *SORD* variants co-segregating with the disease in four families, respectively (Fig. 1a, b). They were absent in several general population frequency databases, such as the 1000 Genomes Project, Exome Aggregation Consortium (ExAC), and the Genome Aggregation Database (gnomAD). To ascertain the pathogenicity of these two novel variants, we analyzed them using several bioinformatic algorithms. The p.His135Arg was predicted to be damaging by SIFT (score 0.001), disease-causing by MutationTaster (score 1.0), probably damaging by PolyPhen-2 (score 1.0), and damaging by CADD (score 24.7). The c.908 + 1 G > C variant was located within the highly conserved donor splice site of exon 8 and predicted to cause abnormal splicing by abolishing the donor splice site of exon 8 (dbscSNV ada-score = 0.9999, rf-score = 0.934).

**Fig. 1:**
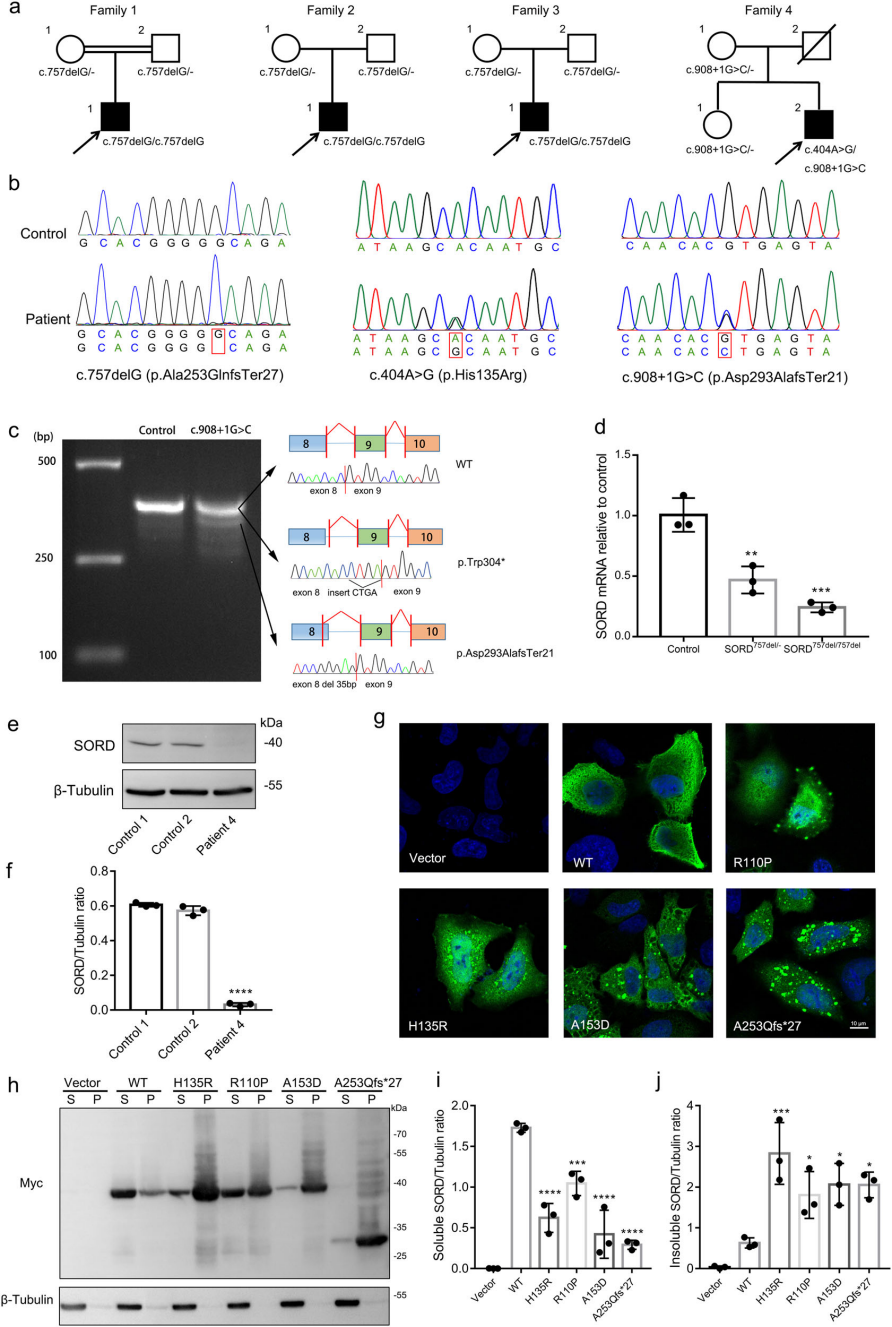
Genetic and functional findings in dHMN patients with *SORD* variants. **a** Pedigrees of four Chinese dHMN families carrying *SORD* variants. Circle: females; square: males; open symbol: unaffected; filled symbol: affected; arrow: proband of each family. **b** Sequencing chromatograms of three variants within *SORD*. The lower chromatogram represents the variant and the upper one represents the normal sequence. **c** *SORD* cDNA products were separated by agarose gel electrophoresis and confirmed by Sanger sequencing. Lane 1: marker; lane 2: control; lane 3: subject II-2 (family 4). **d** Relative *SORD* mRNA expression levels from peripheral blood in subjects with a heterozygous or homozygous c.757delG (p.Ala253GlnfsTer27) compared with the healthy controls (*n* = 3 experimental repeats, data are shown as mean ± SD. **p < 0.01; ***p < 0.001). **e** Fibroblast SORD protein expression levels in two normal controls and patient 4 with compound c.908 + 1 G > C/p.His135Arg variants. **f** The graph shows β-tubulin normalized SORD expression levels (*n* = 3 biological repeats, data are shown as mean ± SD. ****p < 0.0001). **g** Immunofluorescence analysis of HeLa cells expressing WT or mutant SORD. Cells were stained by mouse anti-Myc antibody followed by anti-mouse Alexa Fluor 488 (green)-conjugated secondary antibody and DAPI (blue). Scale bars = 10 μm. **h** Soluble (S) and insoluble (P) protein fractions per variant with western blot probing for Myc-SORD and β-Tubulin (loading control). The graphs show β-tubulin normalized Myc-SORD expression levels per variant in soluble protein fractions (**i**) and insoluble protein fractions (**j**) (*n* = 3 biological repeats, data are shown as mean ± SD. *p < 0.05; ***p < 0.001; ****p < 0.0001). All blots were derived from the same experiment and were processed in parallel.

To confirm whether the c.908 + 1 G > C variant affects splicing, total RNA was isolated from patient’s peripheral blood, and reverse transcribed into cDNA and amplified. Agarose gel electrophoresis showed a normal-sized band and a shorter band in the proband (II-2) of family 4. TA clone and Sanger sequencing of cDNA polymerase chain reaction (PCR) products revealed two abnormal spliced *SORD* transcripts, an amplicon with four nucleotides inserted prior to exon 9 due to the utilization of a cryptic intronic donor splice site (p.Trp304*), and an amplicon skipping of 35 base pairs at the 3′ end of exon 8 that caused premature stop codon p.Asp293AlafsTer21 (Fig. 1c).

To explore the influence of the variant c.757delG (p.Ala253GlnfsTer27) on the function of *SORD* gene, we used quantitative PCR to examine *SORD* mRNA levels in peripheral blood from the proband of family 2 (II-1), his father (I-2), and the healthy control. Compared to the healthy control, the patient carrying a homozygous c.757delG (p.Ala253GlnfsTer27) variant showed a significant reduction in *SORD* mRNA expression levels of ~70–80% when normalized to the control gene *GAPDH* (*p* < 0.001). His father carrying a heterozygous c.757delG (p.Ala253GlnfsTer27) variant exhibited a milder decrease of ~45–50% in mRNA levels (*p* < 0.01; Fig. 1d). It suggested that mRNAs of patients with the c.757delG (p.Ala253GlnfsTer27) variant were unstable on account of the nonsense-mediated mRNA decay (NMD).

We used fibroblast of patient 4 with compound c.908 + 1 G > C/p.His135Arg variants as a model to assess the effects of two variants on SORD protein expression. Western blot showed that SORD protein expression was significantly decreased compared to two healthy controls (Fig. 1 e, f). A previous study has demonstrated that residues 90–110 and 120–140 in SORD subunit are important for the stability and binding of tetramer^7^. To define whether the variant p.His135Arg and two previously reported missense variants (p.Arg110Pro and p.Ala153Asp) flanking it altered the tetramer-forming interactions, five plasmids expressing wild type (WT), R110P, H135R, A153D, or A253Qfs*27 protein were constructed. Immunofluorescence experiment in HeLa cells transiently expressing WT or mutant SORD showed that WT SORD displayed diffuse intracellular staining, whereas mutant SORD displayed granules throughout the cytosol (Fig. 1g). To confirm the presence of SORD aggregates, we investigated the soluble and insoluble SORD fractions by western blot. The levels of mutant SORD protein were markedly reduced in the soluble fractions of transfected cells with an increase in the insoluble fractions (Fig. 1h–j). Altogether, these studies provided evidence that p.His135Arg caused aggregate forming of SORD due to probable conformational changes in tetrameric state, which indicated the variant was pathogenic.

Altogether, we identified 4 unrelated dHMN patients harboring biallelic *SORD* pathogenic variants. The detailed clinical features and nerve conduction studies of these patients were shown in Table 1. All four cases were sporadic and only one (Family 1: II-1) was born of a consanguineous marriage. The average age of disease onset was 12.5 ± 3.5 years (range 9–16). All affected individuals complained about muscle weakness in their lower limbs during their teenage years, thus having difficulty in walking, running, and squatting down. The disease presented slowly progressive in all the probands. The distal muscle atrophy began in their feet and legs, followed by intrinsic muscles of the hands. As a consequence, they were not able to make fine and complicated movements using their hands. On electrophysiological examination, the nerve conduction studies showed length-dependent axonal motor neuropathy with marked decrease in the amplitudes of compound muscle action potentials and mild reduce in the motor nerve conduction velocities in lower limb nerves. The sensory nerve conduction velocities and sensory nerve action potentials were in the normal range.

**Table 1:** Clinical features of patients carrying *SORD* variants.

Family	1	2	3	4
Genotype	c.757delG (p.Ala253GlnfsTer27)	c.757delG (p.Ala253GlnfsTer27)	c.757delG (p.Ala253GlnfsTer27)	c.908 + 1 G > C/c.404 A > G (p.His135Arg)
Sex/age (years)	Male/27	Male/29	Male/30	Male/36
AAO (years)	15	9	10	16
Initial symptoms	Difficulty walking and distal weakness	Difficulty walking and distal weakness	Muscle weakness and atrophy of lower limbs	Difficulty walking
Foot deformity	Bilateral	Bilateral	Bilateral	Bilateral
Limb weakness UL (MRC)	Distal:3	Distal:4	Distal:3	Distal:4
Limb weakness LL (MRC)	Distal:2	Distal:3	Distal:2	Distal:4
Muscle atrophy UL	Distal: moderate	Distal: moderate	Distal: moderate	Distal: moderate
Muscle atrophy LL	Distal: moderate	Distal: moderate	Distal: severe	Distal: moderate
Pinprick sensation	Normal	Normal	Normal	Normal
Knee/ankle DTRs	++/absent	++/++	++/absent	++/++
Age at NCS (years)	17	29	19	31
CMT type	dHMN	dHMN	dHMN	dHMN
Median MCV (m/s)	57.1	NA	56.4	57.3
Median CMAP (mV)	11.5	NA	2.6	3.1
Ulnar MCV (m/s)	66.1	NA	53.0	NA
Ulnar CMAP (mV)	7.3	NA	1.7	NA
Tibial MCV (m/s)	44.8	44.1	48.7	49.3
Tibial CMAP (mV)	2.4	1.8	0.5	6.6
Peroneal MCV (m/s)	40.7	45.0	41.6	49.3
Peroneal CMAP (mV)	0.6	1.3	0.5	1.9
Superficial peroneal SCV (m/s)	48.2	50.0	51.7	48.7
Superficial peroneal SNAP (μV)	13.0	14.0	14.0	15.0
Sural SCV (m/s)	59.3	54.0	49.0	NA
Sural SNAP (μV)	13.0	52.6	19.0	NA

*AAO* age at onset, *UL* upper limbs, *LL* lower limbs, *MRC* Medical Research Council scale, *DTR* deep tendon reflex, *NCS* nerve conduction studies, *CMAP* compound muscle action potential, *MCV* motor nerve conduction velocity, *SCV* sensory nerve conduction velocity, *SNAP* sensory nerve action potential, *NA* not available.

The SORD catalyzes the interconversion between glucose and fructose via sorbitol together with aldose reductase. The polyol pathway is an important alternate route for sugar metabolism. Therefore, it is believed that *SORD* is involved in the development of diabetic neuropathy^8,9^. To date, eight pathogenic variants in *SORD* have been identified in CMT2 or dHMN. The variant c.757delG (p.Ala253GlnfsTer27) is the most common cause with a frequency of up to ~10% in undiagnosed CMT2 or dHMN cases^6^. Similarly, the most common pathogenic variant (c.757delG, p.Ala253GlnfsTer27) was identified in three dHMN families in our study, which occupied 33.3% (3/9) of genetically undefined dHMN cases. In addition, the patient carrying a homozygous c.757delG (p.Ala253GlnfsTer27) variant showed a dramatic reduction in *SORD* mRNA levels. This result was consistent with the finding of the protein expression level in the patient with the c.757delG (p.Ala253GlnfsTer27) variant^6^, indicating a NMD mechanism and loss of function of *SORD* gene. The absence of SORD protein and an increased level of intracellular sorbitol were found in patient-derived fibroblasts^6^. Furthermore, synaptic degeneration and progressive motor impairment appeared in *Drosophila* with loss of *SORD* orthologs^6^.

In conclusion, we identified *SORD* pathogenic variants in four dHMN families, further establishing *SORD* as a disease-causing gene for dHMN. Our findings provide different mutation types of *SORD*, expanding the spectrum of pathogenic variants in *SORD* and highlighting the importance of screening *SORD* variants among undefined hereditary neuropathy patients.

## Methods

### Subjects

A cohort of 20 CMT2, 9 dHMN patients, and available unaffected relatives were enrolled consecutively in this study between June 4, 2008 and October 6, 2019 from southeastern China. They have been excluded other inherited peripheral neuropathies by genetic screening of the known causative genes in our previous reports^10–13^. Each patient received clinical evaluation and diagnosis from at least two senior neurologists, according to clinical features and electrophysiological studies described in our previous reports^11,14^. In addition, the criteria for the enrollment were as follows: (1) autosomal recessive or sporadic; (2) slowly progressive weakness and atrophy of the distal limb muscles; and (3) electrophysiological results showed preserved or only mildly slowed nerve conduction velocities (>38 m/s) and showed primarily axonal damage (CMT2) or a pure motor axonal neuropathy without sensory involvement (dHMN). Written informed consents were obtained from all participants. The study was approved by the ethics board of Second Affiliated Hospital, Zhejiang University School of Medicine, and Huashan Hospital of Fudan University.

### Whole-exome sequencing and bioinformatic analyses

Genomic DNA of the probands and unaffected family members were extracted from peripheral blood using a standard kit (Qiagen, Germany). Then the Agilent SureSelect^TM^ Human All Exome V6 kit (Agilent Technologies Inc, Canada) on an Illumina Hiseq X Ten Analyzer (Illumina, USA) was used to perform WES in all the subjects. The frequency of all the variants in the general population was filtered by the 1000 Genomes Project (https://www.ncbi.nlm.nih.gov/variation/tools/1000 genomes/)^15^, ExAC (http://exac.broadinstitute.org/)^16^, and gnomAD (http://gnomad-old.broadinstitute.org/)^17^. The pathogenicity of the identified variants was analyzed using SIFT (http://sift.jcvi.org/)^18^, PolyPhen-2 (http://genetics.bwh.harvard.edu/pph2/)^19^, MutationTaster (http://www.Mutation taster. org/)^20^, and CADD^21^. To predict the splicing effects of c.908 + 1 G > C variant, two prediction scores (ada-score and rf-score) were extracted from dbscSNV database^22^. The variant is interpreted as splice-altering if the score is >0.6.

### Sanger sequencing of *SORD* and *SORD2P*

Exonic and intronic areas of *SORD* and exon 7 of *SORD2P* were amplified by PCR followed using the primers described previously^6^. Sanger sequencing was performed to validate the candidate variants analyzed by WES and co-segregation of the pedigrees among all available family members.

### RNA isolated, splicing analysis, and quantitative PCR

Total RNA was isolated from peripheral blood treated with RNAiso Plus (Takara, Japan). RNA was reverse transcribed into cDNA using the PrimeScript^TM^ II 1st Strand cDNA Synthesis Kit (Takara, Japan). For the analysis of the splicing variant, PCR products of cDNA were visualized in agarose gels to study the transcribed pattern and then cloned into pGM-simple-T TA vector (TIANGEN, China). The vector was transformed into DH5α cells (TIANGEN, China), and individual colonies were examined and sequenced. The targeted and control region were amplified respectively using the following primers: *SORD* forward: 5′-CAAGCCCAACAACC TTTCCC-3′, *SORD* reverse: 5′-TGAGGGCAGAGCCCATTAAC-3′, *GAPDH* forward: 5′-GGAGCGAGATCCCTCCAAAAT-3′; and *GAPDH* reverse: 5′-GGCTGTTGTCATACT TCTCATGG-3′. The quantitative PCR was run and analyzed on an ABI StepOne real-time PCR system (Thermo Fisher, USA).

### Expression plasmids

The WT full-length coding region of human SORD cDNA (NM_003104.6) was cloned into pCMV-Myc-N terminal tag vector (Clontech, USA). Three missense SORD variants (p.Arg110Pro, p.His135Arg, and p.Ala153Asp) and the nonsense p.Ala253GlnfsTer27 variant reported previously were introduced into WT SORD construct, using PCR mutagenesis (TOYOBO, China).

### Cell culture and plasmids transfection

Primary fibroblasts were obtained from skin biopsy of patient 4 and two normal controls and grown in Dulbecco’s Modified Eagle Medium (Gibco, USA) supplemented with 10% fetal bovine serum (Gibco, USA) at 37 °C in 5% CO_2_. HEK-293T and Hela cell are from CBTCCCAS (The Cell Bank of Type Culture Collection of Chinese Academy of Sciences). For immunofluorescence analysis, Hela cells were cultured in glass-bottomed 24-well plates and transfected with 500 ng of plasmid DNA, using Lipofectamine 3000 reagent (Invitrogen, USA) for 24 h. For protein expression analysis, HEK-293T cells were cultured in 6 cm dishes and transfected with 4000 ng of plasmid DNA, using Lipofectamine 3000 reagent for 48 h.

### Immunofluorescence analysis

Hela cells transiently transfected with Myc-tagged SORD were cultured in glass-bottomed dishes. Cells were fixed with 4% paraformaldehyde for 20 min, permeabilized, and blocked in PBS containing 0.1% TritonX-100 (Beyotime Biotechnology, China) and 5% bovine serum albumin (Sigma, USA) for 60 min. Cells were then incubated with anti-Myc (1:1000, SAB2702192, Sigma, USA) antibody at 4 °C overnight, followed by secondary anti-mouse IgG Alexa Fluor 488 antibodies (1:1000, Life Technologies, USA). Cell nuclei were then stained with 40, 6-diamidino-2-phenylindole (DAPI; 1:5000, Life Technologies, USA). Fluorescence images were captured by Olympus FV3000 confocal system (Olympus Corporation, Japan).

### Protein solubility assay, SDS–PAGE, and immunoblotting

Forty-eight hours after transfection, transfected cells were lysed in the mild lysis buffer (CelLytic^TM^ M, Sigma, USA). Lysates were centrifuged for 15 min with 12,000 r.p.m. by 4 °C to separate soluble and insoluble fractions. The insoluble fraction was washed three times in the mild lysis buffer and resuspended with ultrasound homogenization. For western blots, 20 µg of protein samples from HEK-293T cells or fibroblasts were separated by 10% SDS–polyacrylamide gel electrophoresis (SDS–PAGE). Specific bands were detected with anti-Myc (1:1000, ab18185, Abcam, China), anti-β-tubulin (1:5000, AC010, Abclonal, China), and anti-SORD (1:1000, ab189248, Abcam, China), respectively.

### Statistical analyses

All the variables were presented as mean ± s.d. The data were compared with a two-tailed Student’s *t* test or a one-way ANOVA using GraphPad Prism (GraphPad Software Inc.). *P* values <0.05 were considered significant.

### Reporting summary

Further information on research design is available in the Nature Research Reporting Summary linked to this article.

## Supplementary information

Supplementary Information

Reporting Summary Checklist

## Data Availability

The data that support the findings of this study are available from the corresponding author upon reasonable request. Whole-exome sequencing data for the patient are deposited in the Sequence Read Archive (SRA) under accession code number PRJNA672732.
