# Supplementary material for: Biallelic SORD pathogenic variants cause Chinese patients with distal hereditary motor neuropathy
Source: NPJ Genom Med. 2021 Jan 4;6:1. doi: 10.1038/s41525-020-00165-6 (PMC7782788; doi:10.1038/s41525-020-00165-6)
Supplement: Supplementary file 1 — Supplementary Information [file 41525_2020_165_MOESM1_ESM.pdf]

Supplementary Information

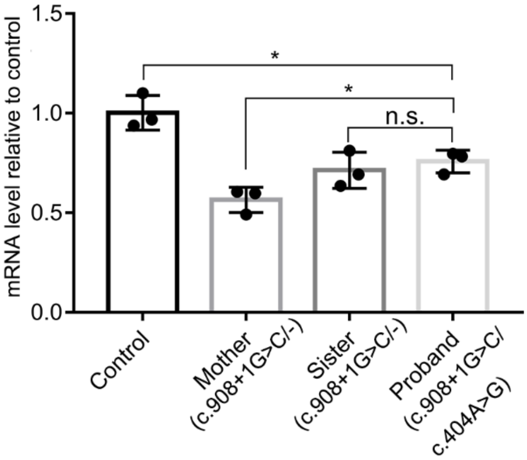

Supplementary Figure 1 Quantitative analysis of SORD transcript level in the proband of family 4 (c.908+1G>C / c.404A>G), his mother (c.908+1G>C/-) and sister (c.908+1G>C/-). Data are shown as means ± SD (n=3 biological repeats). *p<0.05; n.s. = not significant.

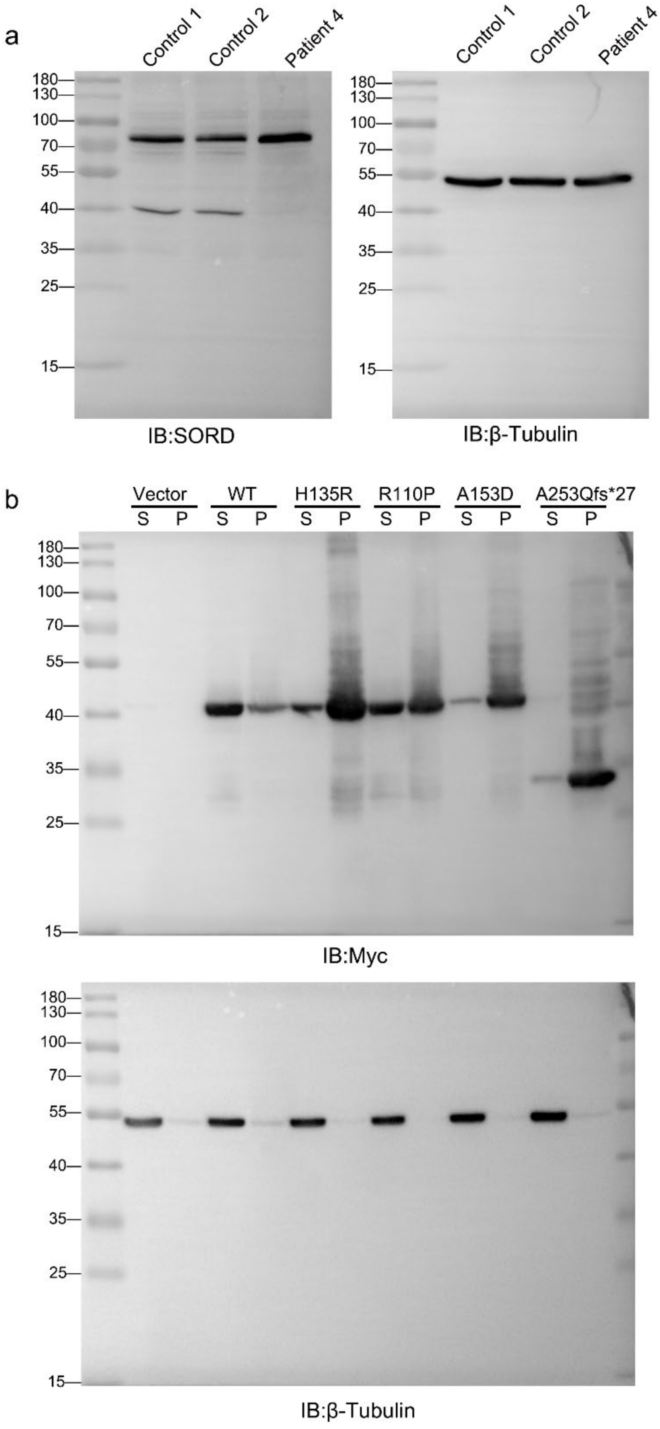

Supplementary Figure 2 (a) Full-length uncropped blots of SORD and β-Tubulin protein from fibroblasts. (b) Full-length uncropped blots of Myc-tagged SORD and β-Tubulin protein from HEK293T cells.
