# Supplementary material for: Biallelic SORD pathogenic variants cause Chinese patients with distal hereditary motor neuropathy
Source: NPJ Genom Med. 2021 Jan 4;6:1. doi: 10.1038/s41525-020-00165-6 (PMC7782788; doi:10.1038/s41525-020-00165-6)
Supplement: Supplementary file 2 — Reporting Summary Checklist [file 41525_2020_165_MOESM2_ESM.pdf]

nature research

Corresponding author(s): DBPR NPJGENMED-00604-T

Last updated by author(s): Oct 27, 2020

Reporting Summary

Nature Research wishes to improve the reproducibility of the work that we publish. This form provides structure for consistency and transparency in reporting. For further information on Nature Research policies, see our Editorial Policies and the Editorial Policy Checklist.

Statistics

For all statistical analyses, confirm that the following items are present in the figure legend, table legend, main text, or Methods section.

n/a	Confirmed	
☐	☒	The exact sample size (n) for each experimental group/condition, given as a discrete number and unit of measurement
☒	☐	A statement on whether measurements were taken from distinct samples or whether the same sample was measured repeatedly
☒	☐	The statistical test(s) used AND whether they are one- or two-sided<br>*Only common tests should be described solely by name; describe more complex techniques in the Methods section.*
☒	☐	A description of all covariates tested
☒	☐	A description of any assumptions or corrections, such as tests of normality and adjustment for multiple comparisons
☐	☒	A full description of the statistical parameters including central tendency (e.g. means) or other basic estimates (e.g. regression coefficient) AND variation (e.g. standard deviation) or associated estimates of uncertainty (e.g. confidence intervals)
☒	☐	For null hypothesis testing, the test statistic (e.g. F, t, r) with confidence intervals, effect sizes, degrees of freedom and P value noted<br>*Give P values as exact values whenever suitable.*
☒	☐	For Bayesian analysis, information on the choice of priors and Markov chain Monte Carlo settings
☒	☐	For hierarchical and complex designs, identification of the appropriate level for tests and full reporting of outcomes
☒	☐	Estimates of effect sizes (e.g. Cohen's d, Pearson's r), indicating how they were calculated

Our web collection on statistics for biologists contains articles on many of the points above.

Software and code

Policy information about availability of computer code

Data collection	no software used
Data analysis	GraphPad Prism 7

For manuscripts utilizing custom algorithms or software that are central to the research but not yet described in published literature, software must be made available to editors and reviewers. We strongly encourage code deposition in a community repository (e.g. GitHub). See the Nature Research guidelines for submitting code & software for further information.

Data

Policy information about availability of data

All manuscripts must include a data availability statement. This statement should provide the following information, where applicable:

- Accession codes, unique identifiers, or web links for publicly available datasets
- A list of figures that have associated raw data
- A description of any restrictions on data availability

The data that support the findings of this study are available from the corresponding author upon reasonable request. Whole-exome sequencing data for the patient are deposited in the Sequence Read Archive (SRA) under accession code number PRJNA672732.

Field-specific reporting

Please select the one below that is the best fit for your research. If you are not sure, read the appropriate sections before making your selection.

☒ Life sciences ☐ Behavioural & social sciences ☐ Ecological, evolutionary & environmental sciences

For a reference copy of the document with all sections, see nature.com/documents/nr-reporting-summary-flat.pdf

Life sciences study design

All studies must disclose on these points even when the disclosure is negative.

Sample size	A cohort of 20 CMT2, 9 dHMN patients, and available unaffected relatives were enrolled consecutively in this study between June 4, 2008 and October 6, 2019 from southeastern China.
Data exclusions	They have been excluded other inherited peripheral neuropathies by genetic screening of the known causative genes in our previous reports
Replication	All in vitro cell functional experiments were repeated three times independently
Randomization	this is not relevant to my study
Blinding	this is not relevant to my study

Reporting for specific materials, systems and methods

We require information from authors about some types of materials, experimental systems and methods used in many studies. Here, indicate whether each material, system or method listed is relevant to your study. If you are not sure if a list item applies to your research, read the appropriate section before selecting a response.

Materials & experimental systems

n/a	Involved in the study	
☐	☒	Antibodies
☐	☒	Eukaryotic cell lines
☒	☐	Palaeontology and archaeology
☒	☐	Animals and other organisms
☐	☒	Human research participants
☒	☐	Clinical data
☒	☐	Dual use research of concern

Methods

n/a	Involved in the study	
☒	☐	ChIP-seq
☒	☐	Flow cytometry
☒	☐	MRI-based neuroimaging

Antibodies

Antibodies used	Anti-Myc tag (SAB2702192) GT0002 Mouse Sigma;<br>Anti-Myc tag (ab18185) Myc.A7 Mouse Abcam ;<br>Anti-Sorbitol Dehydrogenase (ab189248) EPR15857 Rabbit Abcam;<br>β-Tubulin Mouse mAb (AC010) Abclonal
Validation	Anti-Myc tag (SAB2702192) GT0002 Mouse Sigma, citation:Science advances. 2020 Apr 10;6(15):eaay3511. doi: 10.1126/sciadv.aay3511.<br>Anti-Myc tag KO validation:https://www.abcam.cn/myc-tag-antibody-myca7-ab18185.html?productWallTab=ShowAll<br>Anti-Sorbitol Dehydrogenase, citation:Nature Genetics 2020;52:473–481.https://doi.org/10.1038/s41588-020-0615-4.<br>β-Tubulin Mouse mAb citation:Nature. 2020 May;581(7806):89-93. doi: 10.1038/s41586-020-2231-y

Eukaryotic cell lines

Policy information about cell lines

Cell line source(s)	HEK-293T and Hela cell are from The Cell Bank of Type Culture Collection of Chinese Academy of Sciences
Authentication	two cell lines are authenticated
Mycoplasma contamination	no mycoplasma contamination
Commonly misidentified lines (See ICLAC register)	no

Human research participants

Policy information about studies involving human research participants

Population characteristics	A cohort of 20 CMT2, 9 dHMN patients
Recruitment	A cohort of 20 CMT2, 9 dHMN patients, and available unaffected relatives were enrolled from Second Affiliated Hospital, Zhejiang University School of Medicine and Huashan Hospital of Fudan University.
Ethics oversight	The study was approved by the ethics board of Second Affiliated Hospital, Zhejiang University School of Medicine and Huashan Hospital of Fudan University.

Note that full information on the approval of the study protocol must also be provided in the manuscript.
